# Ethanol-extracted Cameroonian propolis exerts estrogenic effects and alleviates hot flushes in ovariectomized Wistar rats

**DOI:** 10.1186/s12906-017-1568-8

**Published:** 2017-01-21

**Authors:** Stéphane Zingue, Chantal Beatrice Magne Nde, Thomas Michel, Derek Tantoh Ndinteh, Jules Tchatchou, Moïse Adamou, Xavier Fernandez, Fernand-Nestor Tchuenguem Fohouo, Colin Clyne, Dieudonné Njamen

**Affiliations:** 1 Laboratory of Physiology and Natural Products Research, Department of Life and Earth Sciences, Higher Teachers’ Training College, University of Maroua, P.O. Box 55, Maroua, Cameroon; 2 Laboratory of Animal Physiology, Department of Animal Biology and Physiology, Faculty of Science, University of Yaounde 1, P.O. Box 812, Yaoundé, Cameroon; 3 Hudson Institute of Medical Research, 27-31 Wright Street, Clayton, VIC 3168 Australia; 4 Institute of Chemistry of Nice, Faculty of Science, University Côte d’Azur, UMR CNRS 7272, Valrose Park, Nice, France; 5 Department of Applied Chemistry, Faculty of Sciences, University of Johannesburg, P.O. Box 17011, Doornfontein, 2028 South Africa; 6 Department of Biological Sciences, Faculty of Science, University of Ngaoundere, P.O. Box 454, Ngaoundere, Cameroon

**Keywords:** Cameroonian propolis, Ethanolic extract of propolis, Phytoestrogens, Hot flushes, Ovariectomized rat, E-screen assay

## Abstract

**Background:**

Since the biological properties of propolis depend to the plants that can be found in a specific region, propolis from unexplored regions attracts the attention of scientists. Ethanolic extract of Cameroonian propolis (EEP) is used to treat various ailments including gynecological problems and amenorrhea. Since there were no scientific data to support the above claims, the present study was therefore undertaken to assess estrogenic properties of Cameroonian propolis.

**Methods:**

To achieve our goal, the ability of EEP to induce MCF-7 cells proliferation in E-screen assay as well as to activate estrogen receptors α (ERα) and β (ERβ) in cell-based reporter gene assays using human embryonic kidney cells (HEK293T) transfected with ERs was tested. Further, a 3-day uterotrophic assay was performed and the ability of EEP to alleviate hot flushes in ovariectomized adult rats was evaluated.

**Results:**

In vitro, EEP showed an antiestrogenic activity in both HEK293T ER-α and ER-β cells. In vivo, EEP induced a significant increase in a bell shape dose response manner of the uterine wet weight, the total protein levels in the uterus, the uterine and vaginal epithelium height and acini border cells of mammary gland with the presence of abundant eosinophil secretions. Moreover, EEP induced a significant decrease in the total number, average duration as well as frequency of hot flushes after 3 days of treatment in rat (equivalent to a month in woman). The dose of 150 mg/kg exhibited the most potent estrogenic effects among all the tested doses. The UPLC-HRMS analysis showed the presence of caffeic acid derivatives and trirtepernoids in EEP, which are well known endowed with estrogenic properties.

**Conclusion:**

These results suggest that Ethanolic extract of Cameroonian propolis has estrogen-like effects in vivo and may alleviate some menopausal problems such as vaginal dryness and hot flushes.

**Graphical abstract:**

Ethanol-extracted Cameroobian propolis exhibited in vitro and in vivo estrogen-like effects. This extract may contain promising phytoestrogens.
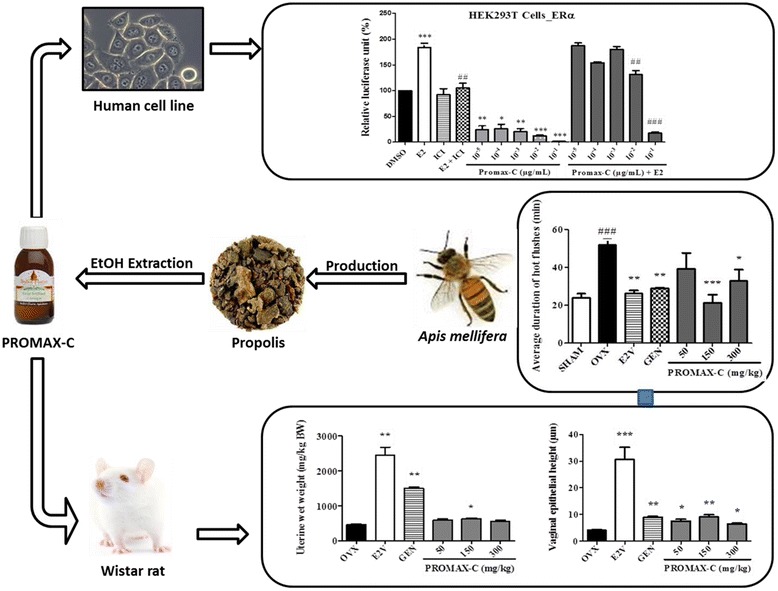

## Background

Hot flushes are the most disturbing and annoying symptom associated with natural menopause or following oophorectomy [1]. Also known as vasomotor symptoms, it occurs in 80% of menopausal women [2]. Hot flush is characterized by a sudden sensation of heat or burning starting in the head or neck and passing over the entire body. Hot flushes are not life-threatening but can negatively affect life’s quality for many women by causing sleep disturbances that often result in fatigue, irritability, forgetfulness, and acute physical discomfort, with negative effects on daily activities and work [3]. In addition, hot flushes may be associated with serious medical conditions. Women with hot flushes may have an increased risk of developing Alzheimer’s disease, a serious neurodegenerative disease, compared to women without hot flushes [4]. Hormone Replacement Therapy (HRT) was for a long time the treatment of choice for the management of climacteric problems [5]. Although estrogen therapy is effective in suppressing hot flushes, it is associated with an increased risk of endometrial cancer, and when combined with a progestin to prevent endometrial hyperplasia, it increases long-term risk of cardiovascular, cerebrovascular, and thromboembolic events [6]. This has resulted in a search for HRT alternatives, and plant-derived substances, so-called phytoestrogens have received a great deal of attention due to its potential protective effects against cardiovascular diseases, osteoporosis and hormone-dependent cancers [7, 8]. These compounds are flavonoids, lignanes, chalcone, coumestane and erythroidine alkaloids [9, 10] and are being increasingly promoted as the safer “natural alternative” to HRT [11].

Propolis is a popular remedy in the folk medicine of several countries and a raw material for numerous preparations, health foods and beverages [12]. It is a strongly adhesive natural mixture produced by honeybees (*Apis mellifera*) from resin collected on buds, leaves and stem barks of some plants, mixed with pollen as well as enzymes secreted from the saliva glands of bees [12–14]. Nowadays, more than 300 compounds, among which flavonoids, terpenoids, steroids, sugars, vitamins and amino acids have been detected in raw propolis and many valuable biological activities have been attributed to propolis [15, 16]. Varied properties of propolis have contributed to its wide use in traditional medical practice and for commercial purposes [12, 13, 17]. Since the biological properties of propolis depend on its chemical composition which greatly varies according to the plants that can be found in a specific region [13, 18, 19], propolis from unexplored regions attracts the attention of scientists in the search of new bioactive molecules [12]. The use of propolis in the treatment and prevention of numerous diseases has been documented [20]. In Cameroon, a natural product prepared as ethanolic extract of propolis is used to treat wounds, burns respiratory and dental infections, stomach ulcer, diabetes, high blood pressure as well as amenorrhea and gynecological problems [21–23]. Although EEP is being used increasingly in Cameroonian traditional system, there are many of its pharmacological activities claimed that remain unproven. The available data on the characteristics of Cameroonian propolis were the work of Mbawala et al. [21, 24] and Seidel et al. [25] on the antimicrobial activities of the ethanol extracts from two different regions (see Table 1). Njintang et al. [23] reported the antiradical activities of Cameroonian propolis. Moreover, there are two reports concerning compounds isolated from Cameroonian propolis [26, 27]. However, there have not yet been reports on estrogenic potential of Cameroonian propolis. In the present study, estrogenic effects of Cameroonian propolis sample collected from Meiganga locality of Adamawa Region of Cameroon were assessed in vitro in E-screen and cell-based reporter gene assays. In vivo, a 3-day uterotrophic assay in ovariectomized adult rats (a classical tool for the detection of estrogenicity of chemicals) was used. Furthermore, its ability to alleviate hot flushes induced in ovariectomized rats was assessed.

**Table 1:** Recapitulative informations on propolis from Adamawa Region, Cameroon

Propolis samples (Year)	Solvant of extraction (Yield)	Phytochemical constituent	Biological activities	References
Propolis Meiganga (2003 & 2005)	Ethanol (3.27%)	Detection of phenolic compounds with HPLC-PDA	Antibacterial activity against gram positive bacteria. Relation between phenolic compounds amount and antibacterial activity.	[21, 24]
Propolis Meiganga (2006)	Methanol (3.7%)	lup-20(29)-en-3-one, lupeol, erythrodiol palmitate, 18-iso-olean-12-ene-3,11-dione, Caffeic acid phenethyl ester (CAPE)	Antinociceptive activity of all the three pentacyclic triterpenoids in the test models of chemical nociception and mechanical hypernociception	[26]
Propolis Meiganga (2008)	Hexane (44.8%)	Alkaloids, Coumarins, Steroids, Triterpenes, Volatile, 2 compound was not yet elucidated	Absence of antibacterial activity	[27]
Propolis Meiganga (2008)	Methanol (3.5%)	Alkaloids, Reducing compounds, Coumarins, Flavonoids, Saponins, Tannins	Antibacterial activities; It was active against *Escherichia coli* and Pseudomonas aeruginosa (MIC: 0.2 mg/ml)	[27]
Propolis Adamawa (2013)	Acetone and methanol (70%)	Terpenoids, phenolic acids, ursolic acid, β-amyrin, Prenylated phloroglucinone, cycloartenol acetate	Phlorogucinonone was found to possess the highest potency against *Trypanosoma brucei brucei*	[52]
Propolis Adamawa (2007)	Ethanol (missing data)	Missing data	Among all African propolis sample tested, Cameroonian propolis was the most potent.	[25]
Propolis Ngaoundal (2011)	Ethanol (5.25%), methanol (9%) and water (1.5%)	Volatile oils, Phenolic compounds, Saponins, Reducing substances, Coumarines, Flavonoids, Triterpenes, Catechic tannins, Fatty acids.	All extracts contain phenolic compounds and present antiradical activities Antioxidant capacities: the order of decreasing antiradical activity is Water > Methanol > Ethanol	[26]
Propolis Ngaoundere (2004)	Ethanol (missing data)	Total polyphenols (mg/L) 10.99 ± 2.56; Tannins (mg/L): 1.57 ± 1.62	The Cameroonians propolis exhibited higher scavenging (antiradical activity (%):83.4 ± 2.3); activity which could justify their commercialisation and role in the management of some chronic diseases	[23]
Propolis Ngaoundere (2003)	Ethanol (missing data)	Total polyphenols (mg/L) 227.8 ± 36.0; Tannins (mg/L): 16.3 ± 12.6 (PROMAX-C, 2003) Total polyphenols (mg/L) 772.8 ± 270.2; Tannins (mg/L): 453.8 ± 361.5 in PROMAX-C of 2006	All PROMAX-C samples tested showed evidence of radical scavenging properties with values ranging from 28 to 70%. Radical scavenging activity: Antiradical activity (%):43.7 ± 13.8 for PROMAX-C made in 2003 and Antiradical activity (%):67.3 ± 3.0 for PROMAX-C made in 2006.	[23]
Propolis Meiganga (2005)	Ethanol (4%)	Contains phenolic compounds	Antibacterial activity against gram positive bacterial strain tested except *Enterococcus faecalis*	[21]
Propolis Martap (2005)	Ethanol (3.5%)	Most active than PROMAX-C from Meiganga with a most higher phenolic content	Antibacterial activity against gram positive bacterial strain tested except *Enterococcus faecalis*. This propolis was the most active and content more phenolic compounds than other tested propolis.	[21]

## Methods

### Chemicals and reagents

Mass Spectroscopy (MS) grade methanol, acetonitrile (ACN), water and formic acid (FA) for UPLC–MS analyses were purchased from Sigma-Aldrich (Saint-Quentin Fallavier, France). Estradiol valerate (Progynova® 2 mg) was purchased from DELPHARM (Lille, France). Genistein was obtained from “Extrasynthese®” (Genay, France). The penicilline (xtapen®) was provided by CSPC Zhongnuo pharmaceutical (Shijiazhuang City, China). The Diclofenac (Dicloecnu®) was provided by ECNU pharmaceutical (Yanzhou City, China). Serums and antibiotics were purchased from GIBCO (Grand Island, NY). The 17β-estradiol benzoate [(Estr-1,3,5(10)-trien-3,16α,17β-triol); purity ≥ 98%] was obtained from Sigma-Aldrich (Hamburg, Germany). The 2-[4-(2-hydroxyethyl)piperazin-1-yl]ethane sulfonic acid (HEPES, purity ≥ 99.5%) was purchased from Ludwig Biotecnologia Ltda (Alvorada, RS, Brazil). Trypan blue, Alamar blue, Sulforodamine B and cell culture mediums were purchased from Sigma-Aldrich (St. Louis, MO, USA). The Smart Button Data loggers were purchased from ACR System Inc (Surrey, Canada).

### Source and preparation of Cameroonian propolis

The propolis used in this study was harvested in Meiganga locality of Adamawa Region in January 2013. The ethanolic extract of propolis was prepared as previously described by Mbawala et al. [21] and Njintang et al. [23] and stored under dry conditions at 4 °C until needed for analysis. Briefly, after drying and grinding, 62.5 g of dried propolis were extracted with 150 mL of ethanol 70% (v/v) at room temperature for 24 h. The ethanol suspension was separate by centrifugation at 1000 rpm for 10 min at room temperature, and the supernatant was poured in a 50 mL dark volumetric flask and the volume completed with 70% ethanol. To achieve our experimental goal, 0.5 L of EEP was lyophilized during 72 h (Christ Beta 1–8 K, Bioblock scientific, Germany) to yield 20.22 g of a brown powder. The extracts were stored under dry conditions at 4 °C until needed for analysis.

### Determination of doses

The doses of administration were calculated based on the posology prescribed for gynecological complaints and amenorrhea: 2 tea spoons in ½ of glass water, 3 times a day. This was powdered by lyophilisation to afford 0.4 g used for one person/day. Taking as average weight per person 70 kg, the extrapolation gave 5 mg/kg BW which was multiplied by 10 to give 50 mg/kg BW considered as pharmacological dose. In order to obtain a dose dependent effect, an intermediate dose of 150 mg/kg and a high dose of 300 mg/kg were obtained by multiplying the low dose by a factor of 3.

### UPLC-HRMS analysis of EEP

The fingerprints were performed using an UPLC Acquity system (Waters, Milford, MA, USA) to ensure a high resolving power and a baseline separation of most of the compounds in a reasonable separation time. All separations were performed on an Acquity UPLC BEH C18 column (100 mm × 2.1 mm I.D., 1.7 μm) at 25 °C with a flow rate of 0.400 mL/min. A guard column (5 mm × 2.1 mm, 1.7 μm) with the same stationary phase was placed before the column. The mobile phase consisted of water + 0.1% FA (solvent A) and ACN + 0.1% FA (solvent B) and was used in multistep gradient mode. The gradient was operated as follow: isocratic 5% B for 0.5 min, 5 to 100% B for 17.5 min, and a final isocratic step for 5 min at 100% B. The sample manager was thermostated at 10 °C, and the injection loop was set at 0.5 μL. The HRMS and HRMS/MS data were acquired with a mass range of 100–1500 m/z using a XEVO-G2QTOF instrument (Waters). ESI conditions operated in negative mode were as follow: source temperature 120 °C, desolvation temperature 500 °C; capillary voltage 1.5 KV, cone voltage 10 V. Nitrogen was used as a cone (10 L/h) and desolvatation gases (1000 L/h). Lockspray flow rate was set at 20 μL/min and lockspray capillary at 2.5 KV. For the HRMS/MS acquisitions, a method including the detection (full scan) and fragmentation of the most intense peaks per scan was used. Collision energy was varying from 10 to 35 V.

### Experimental organisms

#### Cell lines and cell culture

The HEK293T — Human Embryonic Kidney 293 T cells line that contain the SV40 large T-antigen were purchased from ATCC (The Global Bioresource Center, Australia). Luciferase reporter construct was kindly provided by Dr Simon Chu (Hudson Institute of Medical Research, Australia). Cells were transfected using Lipofectamine Reagent obtained from Invitrogen (Sydney, Australia). The MCF7 — human ER-positive breast adenocarcinoma cells was obtained from the Rio de Janeiro Cell Bank (Federal University of Rio de Janeiro, Brazil).

HEK293T cells were cultured routinely in phenol red DMEM-F12 medium containing 10% fetal calf serum (FCS), while MCF-7 cells were cultured in RPMI-1640 medium supplemented with 10% fetal bovine serum (FBS). All cell cultures were also supplemented with 100 U/mL penicillin, 100 μg/mL streptomycin and 10 mM HEPES. The cell cultures were maintained at 37 °C in a 5% CO_2_ humidified atmosphere and pH 7.4. Every two days, cells were passaged by removing 90% of the supernatant and replacing it with fresh medium. In all in vitro experiments, viable cells were checked at the beginning of the experiment by Trypan Blue dye exclusion test.

#### Animals

Healthy juvenile female Wistar rats aged 3 months (∼ 150 g) were obtained from the breeding facility of the Laboratory of Animal Physiology, University of Yaounde I (Cameroon). Animals were housed in clean plastic cages at room temperature (around 25 °C) under natural illumination (approx. 12 h light/dark). They had free access to a standard soy-free rat chow and water *ad libitum*. The composition of animal diet was: corn (36.7%), bone flour (14.5%), wheat (36.6%), fish flour (4.8%), crushed palm kernel (7.3%), sodium chloride (0.3%) and vitamin complex (Olivitazol® - 0.01%).

#### Ethical consideration

Housing of animals and all experiments were approved by the Cameroon Institutional National Ethic Committee, which adopted all procedures recommended by the European Union on the protection of animals used for scientific purposes.

### Study design

#### Cell viability assay

The Cytotoxicity of EEP was evaluated by Alamar Blue (resazurin) assay, in MCF-7 and HEK293T cells. This assay evaluates the mitochondrial production as a measurement of cell viability. For this, a density of 1 × 10^4^ cells/well was seeded in a 96-well plate in 100 μL of culture medium. After 24 h to permit their adhesion, cells were exposed for 24 h to the propolis extract at concentrations ranging from 10^−5^ to 10^−1^ μg/mL and 10^−8^ to 10^−5^ μg/mL for HEK293T and MCF-7 cells, respectively. Each experiment was performed in triplicate and repeated three times.

#### Experiment 1: E-screen assay

The MCF-7 cells proliferation assay was performed as described by Resende et al. [28]. Briefly, cells were trypsinized and seeded in 24-well plates at an initial concentration of 2 × 10^4^ cells per well in RPMI supplemented with 10% FBS. After 24 h of incubation (37 °C, 5% CO_2_) to permit their adhesion, cells were washed with phosphate-buffered saline (PBS) and the Serum Replacement 2 (0.5×) supplemented phenol red-free RPMI was substituted for the seeding medium. EEP was added to the experimental medium at concentrations from 1 × 10^−8^ to 1 × 10^−5^ μg/mL. For antiestrogenicity tests, before incubation, 1 × 10^−8^ M of 17β-estradiol was added to the wells. Cells treated with DMSO (0.01%) and 10% FBS in RPMI were solvent and medium controls, respectively. The steroid-free experimental medium serves as negative control while cells treated with 1 × 10^−8^ M of 17β-estradiol was positive control. The assay was stopped after 144 h by removing the medium from wells, fixing the cells with cold 10% trichloracetic acid and incubated at 4 °C for 1 h. Thereafter, cells were washed four times with tap water and dried. Furthermore, cells were stained during 30 min with 0.057% (w/v) sulforhodamine-B (SRB) dissolved in 1% acetic acid, rinsed four times with 1% acetic acid and air dried. Bound dye was solubilized with 10 mM Tris base (pH 10.5) in a shaker. Finally, aliquots were read in a Biotek EL800 Multiscan apparatus (Winoosky, USA) at 510 nm. The estrogenic activity results were expressed as mean ± standard error of mean (SEM) of the proliferative effect (PE), which was calculated according to Schiliro´et al. [29]: *PE = max cell number of sample/cell number of DMSO control*. The estrogenic activity of a sample was determined as the relative proliferative effect (RPE%). The RPE compares the maximum proliferation induced by a sample with that induced by 17β-estradiol: *RPE% = [PE for sample/PE for 17β-estradiol]* × *100* [28].

#### Experiment 2: transfections and luciferase assays

The ability of EEP to activate α and β estrogen receptors, in cell-based assays was tested. The Human Embryonic Kidney 293 T cells (HEK293T) were transiently transfected as previously described by Zingue et al. [30]. They were then treated with different concentrations (from 10^−5^ to 10^−1^ μg/mL) of EEP for 24 h. Cells treated with E2 alone served as positive control. Reporter gene assays in HEK293T-ERα cells and HEK293T-ERβ cells were performed using a commercial kit (Promega, Australia) according to the manufacturer’s instructions. Luciferase activity was measured and normalised against β-galactosidase activity determined by using the 2-nitrophenyl β-D-galactopyranoside (ONPG) method (Sigma-Aldrich, Sydney, Australia). Each experiment was performed at least in duplicate and repeated three times.

#### Experiment 3: the 3-day uterotrophic assay

Estradiol valerate, genistein and EEP were dissolved in distilled water (dH_2_O) used as vehicle in this experiment. Thirty female Wistar rats received a single intramuscular dose of long acting penicillin and diclofenac (10 mg/kg and 3 mg/kg respectively) the day before ovariectomy. Thereafter they were bilaterally ovariectomized (OVX) using the dorsal approach under Diazepam and ketamin anesthesia (respectively 10 mg/kg and 50 mg/kg BW; *i.p.*). Fourteen days after ovariectomy (time necessary for endogenous hormonal decline), animals were randomly distributed into 6 groups of five animals each (*n* = 5) and treated once daily for 3 consecutive days by gavage with 10 mL/kg of distilled water (OVX), 1 mg/kg of estradiol valerate (E2V) and 10 mg/kg of genistein (GEN). The remaining three groups received EEP at doses of 50, 150 and 300 mg/kg BW. Twenty four hours after the last administration, animals were sacrificed by decapitation. Uteri were collected, trimmed of fat and wet weighed. Uterus, vagina, and mammary gland were fixed in 10% formalin for histological analyses. Estrogenic effects were evaluated based on uterine wet weight, the uterine and vagina epithelial heights, total uterine protein levels and mammary gland differentiation.

#### Experiment 4: measurement of hot flushes

The measurement of hot flushes have been made as previously described by Zingue et al. [30]. Data loggers were used to monitor the core temperature changes in the animals at 2 min intervals for 72 h and were preset to start measuring core temperatures 12 h before the beginning of the treatment until the end of treatment. A total of 35 acclimatized female rats were used in this experiment. A 4-cm long skin and abdominal musculature incisions were made in the cote region of abdomen under valium and ketamin anesthesia (respectively 10 and 50 mg/kg BW; *i.p.*). A data logger protected in sterilized neutral wax was placed in the abdominal cavity. Animals of group 1 (*n* = 6) were considered as control sham-operated (Sham) in which, the ovaries were exposed and gently manipulated but not excised and the other 30 animal were ovariectomized (OVX) as described above. The abdomen was closed with absorbable simple interrupted sutures in the muscle layer and skin. Animals of group 1 received distilled water as vehicle, while the 30 ovariectomized rats were randomly distributed into 6 groups of 5 animals each (*n* = 5) and treated for 3 days as described above. Twenty four hours after the last administration animals were sacrificed by decapitation, and the data loggers recovered. Data (central body temperature) was retrieved from loggers unto Excel spreadsheets and analyzed using the ACR Trend Reader for Smart Button Software. Substances were evaluated for their ability to affect core temperature; an average core temperature was calculated after every 6 h time point. The mean core temperature change (Δ core temperature) was determined as previously described by Maswood et al. [31]. Hot flushes were considered for any internal temperatures ≥ 38 °C. The total number of hot flushes, the average of these hot flush durations and the frequency of hot flushes were determined as described by Zingue et al. [30].

### Histomorphological analysis

The formalin-fixed tissues were embedded in paraffin, and sections of 5-μm thickness were cut. Following hematoxylin-eosin staining, mammary gland differentiation, uterine and vaginal epithelial height were assessed on microphotography using the complete Zeiss equipment consisting of a microscope Axioskop 40 connected to a computer where the image was transferred, and analyzed with the MRGrab 1.0 and Axio Vision 3.1 softwares, all provided by Zeiss (Hallbermoos, Germany).

### Biochemical analysis

Uterine total protein levels were determined in uteri using colorimetric methods described by Gonal et al. [32].

### Statistical analysis

The data from each experimental group (*n* = 5) were expressed as mean ± SEM. All graphs were plotted with Sigma Prism 5.0. One-way analysis of variance (ANOVA) followed by Dunnett’s test for multiple comparisons and the Student’s *t-* test were used for statistical comparison between different control and treated groups for in vivo and in vitro experiments respectively. The significance of the difference was fixed at *p* < 0.05.

## Results

### Phytochemical analysis

**Fig. 1:**
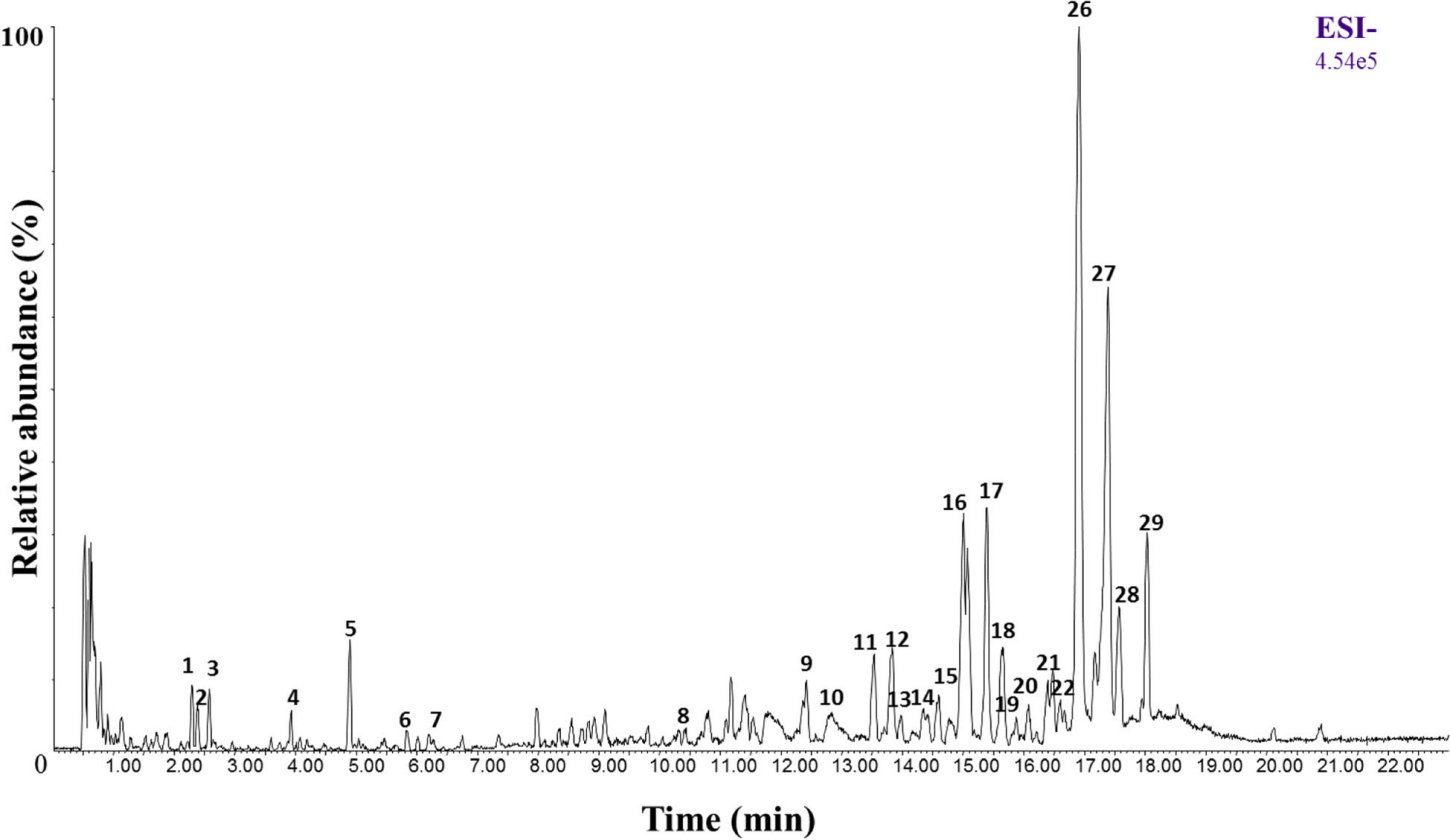
UHPLC–ESI-HRMS base peak chromatogram of EEP in the negative ionisation mode

UPLC-HRMS analysis (Fig. 1 and Table 2) was carried out to identify compound from ethanolic extract of Cameroonian propolis. Briefly, all well resolved peaks in BPI were selected and possible elemental compositions (EC) were calculated. For reducing the possible EC candidates, mass tolerance was set below 3 ppm, only C, H, O and N were selected for calculations and only consistent RDBeq values were considered. Additionally HRMS/MS data as well as bibliographic information were employed to identify the compounds. Furthermore dimeric ions [2 M-H]^−^ observed in case of triterpenoic compounds supported the confirmation of EC for the [M-H]^−^ ion. The detected compounds of PROMAX-C are summarised in Table 2 including their retention time, EC, *m/z* (monoisotopic mass), RDBeq and their major HRMS/MS fragments. Identified metabolites are mainly caffeic acid derivatives and triterpenoids. Indeed, diverse caffeic acid derivatives already described in propolis extracts from Brazil and sub-Saharan African countries [33, 34] have been detected as caffeic acid 4-*O*-arabinoside (*m/z* = 311.0768), caffeic acid 4-O-xyloside (*m/z* 311.0768), caffeoylquinic acid (*m/z* = 353.0878), caffeic acid 4-*O*-glucoside (*m/z* = 341.0868) and 3,4-dimethyl caffeic acid (*m/z =* 207.0656). Herein it was not possible to distinguish caffeic acid 4-*O*-arabinoside from caffeic acid 4-*O*-glucoside that is why they were noted as caffeic acid pentoside. In addition, two others common phenolic compounds in propolis were characterised: coumaric acid (*m/z* = 163. 0386) and phloretic acid (*m/z* = 165.0543) [14].

**Table 2:** Summary of compounds separated and identified in Cameroonian propolis by UHPLC-ESI-HRMS analysis in the negative ion mode. Rt, [M-H]^−^, [2 M-H]^−^, EC and RDBeq information are given together with main fragments at MS/MS level

Pick N°	Tr (min)	[M-H]^−^ (*m/z*)	[2 M-H]^−^ (*m/z*)	EC	Mass errors (ppm)	RDBeq	Fragment ions (m/z)	Tentative identification	References
1	2.30	311.0768		C_14_H_15_O_8_	0.3	7.5	137.0236 93.0335	caffeic acid 4-O-pentoside	[33]
2	2.38	311.0768		C_14_H_15_O_8_	0.6	7.5	137.0236 93.0346	caffeic acid 4-O-pentosside	[33]
3	2.60	341.0868		C_15_H_17_O_9_	1.5	7.5	167.0317	Caffeic acid 4-O-glucoside	[33]
4	2.80	353.0878		C_16_H_17_O_9_	1.4	8.5	191.0558 173.0451	caffeoylquinic acid	[34]
5	3.71	163.0386		C_9_H_7_O_3_	−5.5	6.5		Coumaric acid	[14]
6	6.21	207.0656		C_11_H_11_O_4_	−0.5	6.5		3.4-Dimethyl caffeic acid (DMCA)	[33]
7	6.26	165.0543		C_9_H_9_O_3_	−5.5	5.5		Phloretic acid	[34]
8	10.332	477.3204		C_28_H_45_O_6_	−2.5	6.5	431.3155 415.2839	Terpenoid derivative	
9	12.36	503.3375		C_30_H_47_O_6_	0.4	7.5	487.3423 441.3373	Triterpenoid	
10	12.42	487.3424		C_30_H_47_O_5_	0.2	7.5		Triterpenoid	
11	13.503	473.3275		C_29_H_45_O_5_	1.7	7.5	411.3252 325.1831	Triterpenoid	
12	13.7	469.3315		C_30_H_45_O_4_	−0.6	8.5	415.3215 325.1845	Triterpenoid	
13	13.82	487.3419		C_30_H_47_O_5_	−0.8	7.5	425.3147	Triterpenoid	
14	14.399	485.3278		C_30_H_45_O_5_	2.3	8.5	471.3471 441.3362	Triterpenoid	
15	14.57	487.3427		C_30_H_47_O_5_	0.8	7.5	459.347	Triterpenoid	
16	15.01	485.3278		C_30_H_45_O_5_	0	8.5	433.3362 325.1843	Triterpenoid	
17	15.07	471.3468		C_30_H_47_O_4_	0.5	7.5	453.3362 427.357	Maslinic or corosolic or cycloanostoic acid derivatives	[13, 38]
18	15.374	469.3315		C_30_H_45_O_4_	−0.6	8.5	425.3418	Triterpenoid	
19	15.857	299.2596		C_18_H_35_O_3_	0.3	1.5		3-hydroxystearic acid	
20	16.2	431.3159		C_27_H_43_O_4_	−0.5	6.5	369.3164	Terpenoid derivative	
21	16.40	455.3513	911.7104	C_30_H_47_O_3_ C_60_H_95_O_6_	−2.4 -2.7	7.5 13.5		Mangiferolic or isomangiferolic acids	
22	16.42	501.3576		C_31_H_49_O_5_	−0.8	7.5		Triterpenoid	
23	16.46	469.3313	939.6696	C_30_H_45_O_4_ C_60_H_91_O_8_	−1.1 −2.7	8.5 13.5		Ambolic acid	
24	16.63	453.3368	907.6818	C_30_H_45_O_3_ C_60_H_91_O_6_	−0.2 −0.2	8.5 15.5		Mangiferonic acid	
25	16.91	471.347	943.7007	C_30_H_47_O_4_ C_60_H_95_O_8_	−0.8 −2.1	7.5 13.5	409.3465	Maslinic or corosolic or cycloanostoic acid derivatives	[13, 38]
26	17.17	471.3467		C_30_H_47_O_4_	−0.4	7.5	455.3512 393.3148	Maslinic or corosolic or cycloanostoic acid derivatives	[13, 38]
27	17.38	471.3467		C_30_H_47_O_4_	−0.8	7.5	409.347 393.3148	Maslinic or corosolic or cycloanostoic acid derivatives	[13, 38]
28	17.96	455.3525		C_30_H_47_O_3_	−2	7.5		Mangiferolic or isomangiferolic acids	
29	18.02	457.3676	915.7413	C_30_H_49_O_3_ C_60_H_99_O_6_	−1.3 −2.1	6.5 11.5	457.3644	Ocotillone isomer	[38]

In addition several triterpenoids were well detected in Cameroonian propolis and putative identification was attempted comparing their ECs and fragmentation pathway with those reported in literature and databases (Table 2). The detected triterpenoids exhibit the C_30_ skeleton characteristic of this family, the number of hydrogen atoms was varying from 43 to 50, and the oxygen atom numbers ranged from three to six. Concerning the fragmentation patterns, their HRMS/MS spectra were dominated by typical neutral loss of 18, 28, 44 and 62 which could be assigned to H_2_O, CO, CO_2_ and H_2_O + CO_2_, respectively. It is worthwhile to note that these typical losses have already been observed for triterpenoids fragmentation in negative mode using either ESI or Atmospheric Pressure Photo Ionisation (APPI) sources [35, 36].

Among them, several triterpenoids were detected at m/z = 455.3513 (C_30_H_47_O_3_) and m/z = 471.3468 (C_30_H_47_O_4_) which corresponds to pentacyclic triterpenoids structure. Some pseudo molecular ions could be attributed to triterpenoids already found in Cameroonian propolis [37] such as mangiferolic or isomangiferolic acids (*m/z* = 455.3513), magiferonic acid (*m/z* = 453.3368) and ambolic acid (*m/z* 469.3315), while the ion at m/z = 471.3468 could be originated from maslinic and corosolic acids already detected in Thai propolis [38] or cycloanostoic acid derivatives from Cretan propolis [13]. Sanpa et al. [38] also idenfied two isomers of ocotillone, tetracyclic triterpenoids, which might correspond to the ion found at *m/z* = 457.3676. Compound detected at *m/z* = 485.3278 (C_30_H_45_O_5_) share same molecular formula than (24*E*)-3-oxo-27,28-dihydroxycycloart-24-en-26-oic acid found in propolis from Burma [39]. Studying main fragment ions, it can be observed formation of ion at m/z = 425.3418 (C_30_H_49_O) corresponding to derivatives of amyrin isomers or lupeol mainly present in Cameroonian propolis [37]. However data were not sufficient to confirm the structure of these compounds and differentiate them.

### In vitro estrogenicity assessment

#### Cytotoxicity

**Fig. 2:**
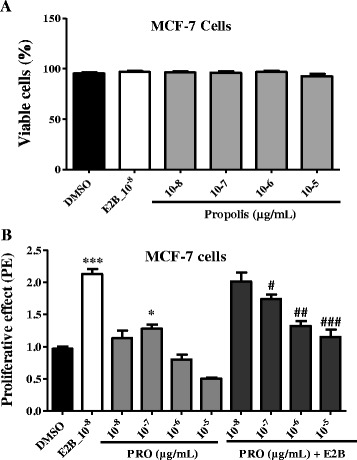
Effects of EEP on MCF-7 cells proliferation. Its effect was investigated by measuring E-screen assay. The relative MCF-7 cells yields (PE) were measured in the presence of DMSO (0.01%), 17β-estradiol (E2B, 10 nM) and EEP (PRO). PE = max cell number of sample/cell number of DMSO control; * *p* < 0.05, *** *p* < 0.001 as compared to the DMSO control

**Fig. 3:**
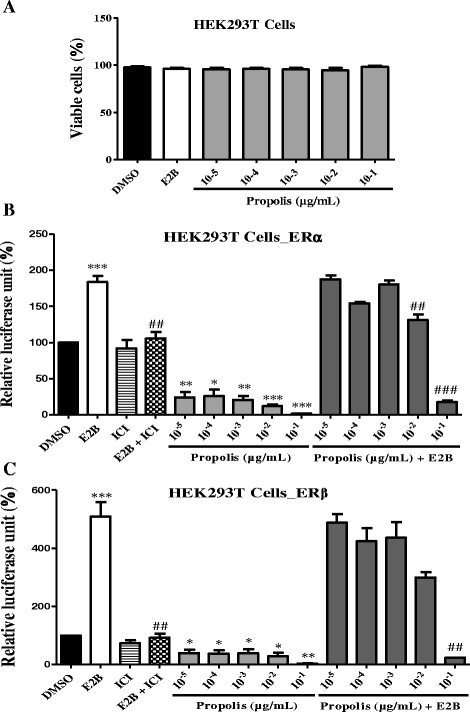
Effects of EEP on the activation of estrogen α and β receptors in HEK293T cells. The effect of EEP on estrogen α and β receptors activity in the transiently transfected HEK293T-ERα and HEK293T-ERβ cells was investigated by measuring reporter gene-coupled luciferase activity. The relative luciferase units (RLU) were measured in the presence of DMSO (0.1%), E2B (10 nM) and Cameroonian propolis; * *p* < 0.05, ** *p* < 0.01, *** *p* < 0.001 as compared with control

Ethanolic extract of propolis did not induced cytotoxic effects in both MCF-7 and HEK293T cells at tested concentrations (Figs. 2a and 3a).

#### E-screen assay

Effects of EEP on MCF-7 cells proliferation are depicted in Table 3 and Fig. 2. It can be observed that 17-β estradiol induced a significant (*p* < 0.001) increased of MCF-7 cells yield. EEP induced a significant (*p* < 0.05) increase of MCF-7 cells yield only at the concentration of 0.1 μM as compared to DMSO control. Further, a significant and concentration-dependant antiestrogenic effect was noted with EEP.

**Table 3:** Effects of EEP in MCF-7 cells proliferation assay

Group	Concentration (μg/mL)	PE	RPE (%)
DMSO	-	1 ± 0.03	47.17
E2B (10^−8^ M)	10^−8^	2.12 ± 0.13	100
Propolis	10^−8^	1.21 ± 0.15	57.07
	10^−7^	1.32 ± 0.18	62.26
	10^−6^	0.72 ± 0.08	33.96
	10^−5^	0.48 ± 0.16	22.64
Propolis + E2B (10^−8^ M)	10^−8^	1.95 ± 0.03	91.98
	10^−7^	1.72 ± 0.19	81.13
	10^−6^	1.31 ± 0.06	61.79
	10^−5^	1.21 ± 0.15	57.07

*DMSO* negative control, *E2B* Estradiol benzoate, served as positive control, *PE* Proliferative effect calculated as the effect on solvent control, *RPE* Relative proliferative effect, compares the maximum proliferation induced by a sample with that induced by 17β-estradiol

#### Transactivation assay

EEP activated ERα and ERβ at all tested doses, but it did not exhibit agonistic activity (Fig. 3). Interestingly when cotreated with E2, the high concentrations (0.01 and 0.1 μg/mL) of EEP significantly antagonized E2-activation of both receptor subtypes.

### In vivo estrogenicity assessment

#### Effects on the uterine wet weight and total protein levels in uterine

**Fig. 4:**
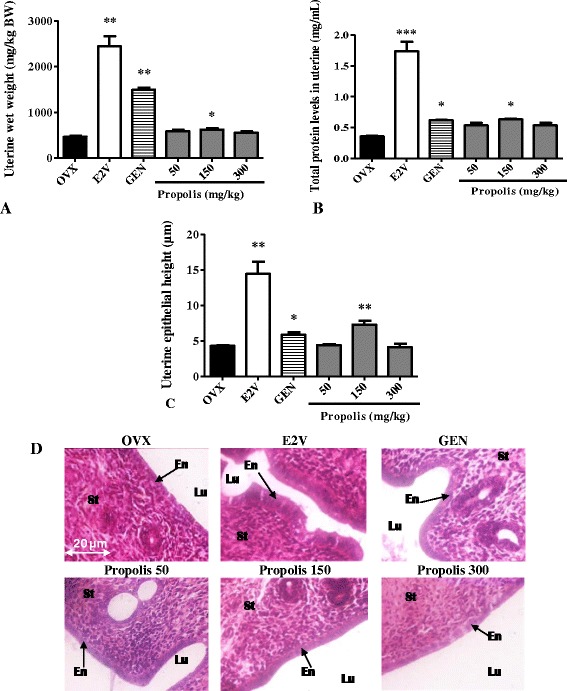
Effects of a 3-day treatment with EEP on the uterine wet weight (**a**), total protein levels in uterine (**b**), uterine epithelial height (**c**) and microphotographs (**d**). OVX = OVX animals treated with the vehicle; E2V = OVX animals treated with estradiol valerate at 1 mg/kg BW; GEN = OVX animals treated with genistein at 10 mg/kg BW; Propolis = OVX animals treated with EEP at doses of 50, 150 and 300 mg/kg BW. *p < 0.05, **p < 0.01 as compared with control. Lu: uterine lumen; En: Endometrium; St: Stroma

As shown in Fig. 4a, a 3-day oral administration of EEP induced a significant increase in the uterine wet weight and total uterine protein levels at all tested doses in a bell shape dose response. The maximum increase for these two parameters was obtained at the dose of 150 mg/kg BW (*p* < 0.01). However, this increase remained much lower than in E2V-treated group.

#### Effects on the uterine epithelium

As depicted in Fig. 4c, following a 3-day treatment with EEP, uterine epithelial height significantly increased by 58.5% (*p* < 0.05) only at dose of 150 mg/kg BW. However these increases remained much lower than that induced by E2V at the dose of 1 mg/kg BW, which showed a 3.5-fold (*p* < 0.01) increase of uterine epithelial height but seems to be higher than those induced by genistein (10 mg/kg). These effects were materialized in histological sections by an atrophic uterus with cuboidal endometrial epithelium and loose connective tissue composed of round nuclei in an unorganized pattern in animals of OVX group. While in the E2V treated-group, all structures are hypertrophic and hyperplastic; the endometrium is multilayered with squamous metaplasia and atypic mitotic figure surrounded by anaplastic epithelial nuclei (Fig. 4d). Microphotographs of animals that received EEP at the dose of 150 mg/kg displayed an endometrium consisting of tall single-layered epithelial cells with abundant mitotic figures and necrosis (arrowhead), however, this effect is less than those observe in E2V-treated group.

#### Effects on the vaginal epithelium

**Fig. 5:**
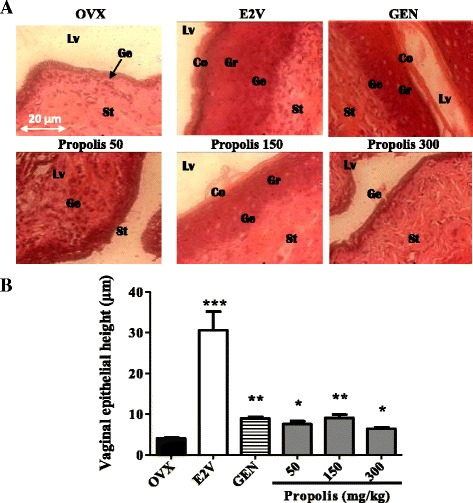
Effects of a 3-day treatment with EEP on the vaginal epithelium: microphotographs (**a**) and epithelial height (**b**). OVX = OVX animals treated with the vehicle; E2V = OVX animals treated with estradiol valerate at 1 mg/kg BW; GEN = OVX animals treated with genistein at 10 mg/kg BW; Propolis = OVX animals treated with EEP at doses of 50, 150 and 300 mg/kg BW. * *p* < 0.05, ** *p* < 0.01 as compared with control. Lv = vaginal lumen, Co = stratum corneum, Gr = stratum granulosum, Ge = stratum germinativum, St: Stroma

Figure 5 represents vaginal epithelial heights. The microphotographs of vaginal epithelium of the OVX group showed an atrophic vaginal epithelium, consisting simply of the stratum germinativum (Ge) which is composed of a few layers of flattened cells (Fig. 5a). After genistein (10 mg/kg) treatment, vaginal epithelium became hypertrophic and hyperplasic (Gr), with cornification (Co) in the upper layers. While E2V (1 mg/kg) induced a stronger hypertrophy and hyperplasia of vaginal epithelium. EEP also induced hypertrophic and hyperplasic effects at all tested doses with cornification at the dose of 150 mg/kg. However compared to E2V there are less cell layers and a lower degree of cornification.

The graphical representation of the vaginal epithelial height (Fig. 5b) shows that E2V induced a 5-fold (*p* < 0.01) increase of vaginal epithelial height. EEP significantly (*p* < 0.01) and in the bell shaped dose response manner increased vaginal epithelial height at all tested doses. The maximum increment of 2.2-fold was obtained at the dose of 150 mg/kg BW (from 4.06 ± 0.21 to 9.47 ± 1.07 μm) as compared to the OVX group.

#### Effects on mammary glands

**Fig. 6:**
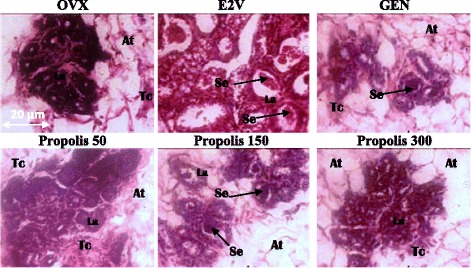
Effects of a 3-day treatment with EEP on mammary gland. OVX = OVX animals treated with the vehicle; E2V = OVX animals treated with estradiol valerate at 1 mg/kg BW; GEN = OVX animals treated with genistein at 10 mg/kg BW; Propolis = OVX animals treated with EEP at doses of 50, 150 and 300 mg/kg BW. La = lumen of alveoli; Ep = aveoli epitheluim; At = adiposite tissue; Se = eosinophil secretion

Figure 6 presents sections of mammary glands. Ovariectomy induced an atrophy of mammary gland which is materialized in OVX-histological section by a modest alveolar development, a small cluster of densely packed epithelial cells without luminal formation are present in the deep subcutaneous fat pad, the loss of the gland parenchyma (Tc) and the ductular and alveolar components, while adipocyte tissue (At) appears prominent. Mammary glands of genistein-treated group depict a few terminal structures with small lumina with secretory material; while mammary glands of E2V-treated group present well-formed acinar and luminal structures, an increase in proliferative activity compared to OVX group such as increase of the diameter and the lumen of alveoli, abundant eosinophil secretion (Se) in lumen of alveoli and further compression of stromal fat. Similar changes were noticed after a 3-days treatment with EEP at all tested doses but only the dose of 150 mg/kg BW presented an eosinophil secretion in their lumens.

### Effects of EEP on hot flushes

#### Effect on core temperature

**Fig. 7:**
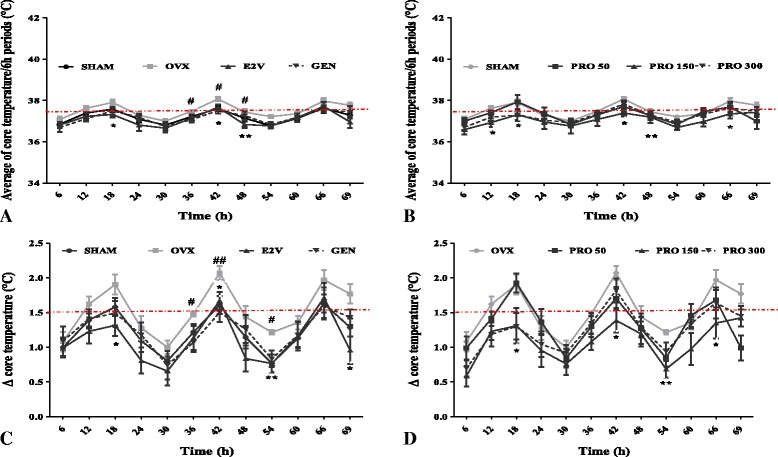
Effects of a 3-day treatment with EEP on mean core temperature (**a & b**) and core temperature changes (**c & d**). SHAM = Sham operated rats treated with the vehicle; OVX = OVX animals treated with the vehicle; E2V = OVX animals treated with estradiol valerate at 1 mg/kg BW; GEN = OVX animals treated with genistein at 10 mg/kg BW; PRO = OVX animals treated with EEP at doses of 50, 150 and 300 mg/kg BW. * *p* < 0.05, ** *p* < 0.01 as compared to control. ^**#**^ *p* < 0.05 as compared to Sham. T = treatment. The red line depicts the normal core temperature and variation of core temperature in rat

In Fig. 7, the result displayed shows that the OVX-untreated group had an average core temperature higher than those of sham operated group and all treated groups (Fig. 7a and b). In this study, core temperature peaked between 10:00 PM and 04:00 AM in all experimental groups. Rats treated with standard drugs (E2V and genistein) had lower temperature peaks compared to the OVX animals (Fig. 7a). Similar changes were observed following treatment with EEP at the dose of 150 mg/kg (Fig. 7b).

Figure 7c, d and Table 4 show the mean core temperature changes. The mean and maximum core temperature changes were significantly higher in OVX group than normal animals (Sham) (*p* < 0.05) (Fig. 7c, d and Table 4). E2V and genistein treatments significantly reduced the core temperature changes as compared to OVX group (*p* < 0.05). Importantly, EEP has significantly reduced the core temperature changes (*p* < 0.05) at the dose of 150 mg/kg) (Table 4).

**Table 4:** Effects of EPP on core temperature changes (Δ)

Groups	Mean Δ Core temperature (°C)	Max Δ Core temperature (°C)
Sham	1.22 ± 0.13	1.63 ± 0.23
OVX	1.5 ± 0.15 #	2.07 ± 0.10 #
E2V	1.1 ± 0.15**	1.71 ± 0.21 *
GEN	1.23 ± 0.12*	1.59 ± 0.17 *
Propolis 50	1.35 ± 0.13	1.60 ± 0.16
Propolis 150	1.09 ± 0.10**	1.40 ± 0.32 **
Propolis 300	1.27 ± 0.12	1.55 ± 0.21*

* *p* < 0.05, ** *p* < 0.01 as compared to OVX control. ^#^ *p* < 0.05, ^##^ *p* < 0.01 as compared to Sham

#### Effects on total number and average duration of hot flushes

**Fig. 8:**
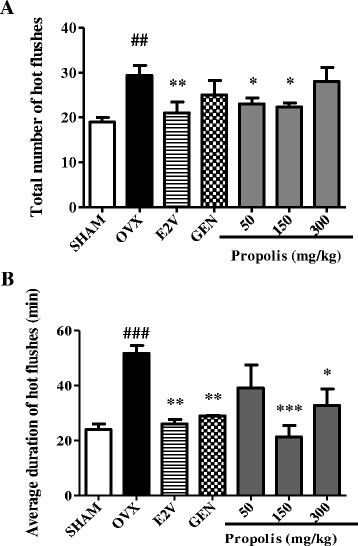
Effects of a 3-day treatment with EEP on total number (**a**) and average duration (**b**) of hot flushes. SHAM = Sham operated rats treated with the vehicle; OVX = OVX animals treated with the vehicle; E2V = OVX animals treated with estradiol valerate at 1 mg/kg BW; GEN = OVX animals treated with genistein at 10 mg/kg BW; Propolis = OVX animals treated with EEP at doses of 50, 150 and 300 mg/kg BW. * *p* < 0.05, ** *p* < 0.01 as compared to control. ^**#**^ *p* < 0.05, ^**##**^ *p* < 0.01, ^**###**^ *p* < 0.001 as compared to Sham

If we consider any core temperatures ≥ 38 °C as hot flushes, ovariectomy significantly increased the total number of hot flushes in OVX animals as compared to normal animals (Sham) (*p* < 0.01). E2V-treatment significantly decreased the total number of hot flushes by 28.5% (from 29.4 ± 2.2 to 21.1 ± 2.42 hot flushes) as compared to OVX group. Moreover, 3-days of oral administration of EEP significantly reduced the total number of hot flushes by 21.8% at doses of 50 mg/kg and by 20.7% at the dose of 150 mg/kg BW (Fig. 8a).

Fourteen days after ovariectomy, the average duration of hot flushes significantly increased in OVX animals as compared to normal animals (Sham) (*p* < 0.001). Treatment with estradiol (E2V) and genistein (GEN) significantly reduced this parameter (*p* < 0.01). The administration of EEP induced a significant (*p* < 0.001) decrease in the average duration of hot flushes only at doses of 150 mg/kg BW (Fig. 8b).

#### Effects on frequency of hot flushes

**Fig. 9:**
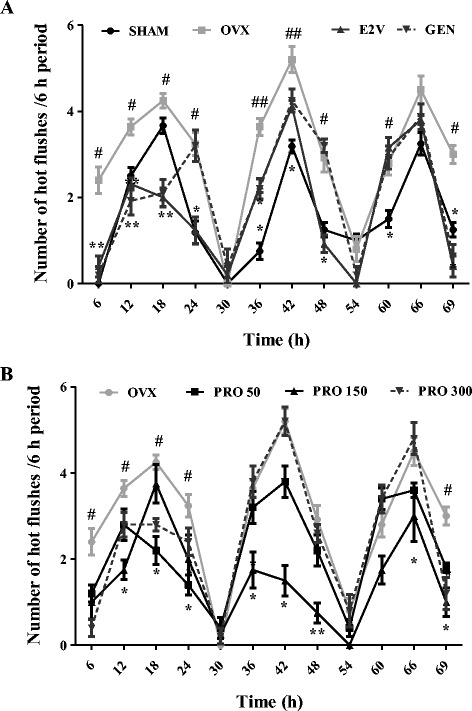
Effects of a 3-day treatment with standard drugs (**a**) and EEP (**b**) on frequency of hot flushes. SHAM = Sham operated rats treated with the vehicle; OVX = OVX animals treated with the vehicle; E2V = OVX animals treated with estradiol valerate at 1 mg/kg BW; GEN = OVX animals treated with genistein at 10 mg/kg BW; PRO = OVX animals treated with EEP at doses of 50, 150 and 300 mg/kg BW. * *p* < 0.05, ** *p* < 0.01 as compared to control. ^**#**^ *p* < 0.05, ^**##**^ *p* < 0.01 as compared to Sham

All the studied groups presented a fluctuation in the number of hot flushes by a 6 h period between high and low values. Ovariectomy significantly increased the frequency of hot flushes at 6 h intervals as compared to normal group (Sham) (*p* < 0.01). More importantly, in the meantime, values in OVX untreated animals were much higher than for Sham operated and all treated groups. As shown in Fig. 9, EEP at the dose of 150 mg/kg BW significantly reduced (*p* < 0.01) the frequency of hot flushes.

## Discussion

Ethanolic extract of Cameroonian propolis is widely used by Cameroonian population in folk medicine for the treatment of multiple health problems, including gynecological complaints and amenorrhea, which has never been assessed scientifically. Previous reports show that, components of propolis were qualitatively and quantitatively variable, depending on the regional plant ecology surrounding the bee hive. As depicted in Table 1, analyses of numerous Cameroonian propolis samples from Adamawa geographical zones display differences on chemical composition, which in turn influences its biological activity. It is well documented that phenolic compounds constitute the most numerous group of propolis components with respect to the quantity and type out of which flavonoids are the most abundant [40]. Many scientific reports on Cameroonian propolis corroborate this assertion and conclude that total polyphenols contents in Cameroonian propolis fall within the range of values reported for propolis from other countries [21, 24, 26, 27]. More precisely, Njintang et al. [23] reported that the polyphenols content in PROMAX-C samples varied from 186 to 1084 mg/L. This high variability of polyphenols amount in different propolis samples has been attributed to the change of regional plants visited by honeybees. The phytochemical analysis performed on Cameroonian propolis sample studied is in agreement with previous reports. We found a large range of polyphenols, specially, caffeic acid derivatives.

Song et al. [15] reported that ethanolic extract of Korean propolis displays estrogenic activity in estrogen-dependent MCF-7 cells, recombinant ER-α, yeast estrogen receptor transcription system and immature female rats and authors concluded that these effects was initiated through estrogen receptors. In this study, EEP induced a weak estrogenic activity in vitro by increasing the MCF-7 cells yield but, it did not induce transactivation of reporter gene activity at all tested doses in both HEK293T ER-α and ER-β cell systems used in this work. However, it seems to possess antiestrogenic activity when increasing concentrations. These results can be explained by the presence in EEP of caffeic acid derivatives, since caffeic acid phenethyl ester (CAPE), an abundant phenolic ester in propolis is well known to exhibit estrogenic activity. Indeed, Jung et al. [16] demonstrated that CAPE is responsible for, among others, of the estrogenic/antiestrogenic effects of propolis. They showed that CAPE is a selective agonist to ER-β, which does not show any estrogenic effect on estrogen receptor-positive breast cancer cells and in immature rat uterine tissue. For these reasons authors claim that CAPE is a potential modulator of the estrogen receptor [16]. Due to the fact that chemical composition of propolis is highly variable mainly due to the variability of plant species growing around the hive [12], the different amount of caffeic acid derivatives in Cameroonian propolis that in Korean propolis can account for its antagonist effects observed in vitro at the tested doses. It has been reported that CAPE preferentially binds to ERβ and that ERβ isoform is involved in anti-proliferative mechanisms [41]. Chemical composition of propolis greatly varies according to the plants that can be found in a specific region [18, 19]. The caffeic acid derivatives has been detected as potential estrogenic components in Cameroonian and Korean propolis, while Okamoto et al. [42] attributed the estrogenic effects of Brazilian propolis to the well-known phytoestrogens Kaempferol, quercetin, naringenin, Biochanin A and formononetin. These aforesaid suggest a regional difference in estrogenic components of propolis.

In this study, ovariectomy induced a decrease of uterine wet weight, uterine and vaginal epithelial height and atrophy of mammary gland. Moreover, it also induced a significantly increase of the number, duration and frequency of hot flushes in ovariectomized rats (OVX) as compared to normal animals (SHAM). As expected, a 3-day consecutive treatment with E2V at the optimal dose of 1 mg/kg and genistein at the dose of 10 mg/kg significantly reversed atrophy in estrogen target organs (uterine, vagina and mammary gland) and hot flushes observed after ovariectomy. EEP induced a significant increase of uterine wet weight, uterine total protein level, uterine and vaginal epithelial height, mammary gland acini and eosinophil secretion in acini in the bell shaped diagram with the maximum effect at the dose of 150 mg/kg. These results are interesting because the growth-stimulatory effect of EEP at the dose of 150 mg/kg reported in this work is comparable to results of a well characterized phytoestrogen genistein (10 mg/kg), suggesting that purification of the active principle of EEP could increase its activity. Additionally the bell shape diagram observed in nearly all assessed parameters suggest a dose-dependent effect of EEP, which is achieved at the optimal dose (150 mg/kg) and probably decreased due to a phenomenon known as “down regulation” of ERs induced by the high dose. As mentioned above, EEP contains phenolic compounds that are known to stimulate uterine growth, uterine and vaginal epithelial height and mammary gland differentiation in a short-term animal studies [43].

Phenolic compounds contained in EEP might bind to ERs in vivo and modulate the expression of many genes, which can account for the increase in total protein level in uterine, marker of uterine cell proliferation [30, 44]. The increase of protein level in the uterus can induce uterine water imbibition as suggested by some authors [44, 45], hence uterine wet weight increases. It is well know that the removal of endogenous estrogen by ovariectomy results in regression of the mammary gland, and that estrogen-like substances reverse this regression [30, 46]. Although the complete pathophysiology of hot flushes is not yet completely understood, their occurrence is assumed to originate in disturbances of the thermo regulatory processes in the hypothalamus, which acts as the body’s thermostat [2]. Fluctuation or decline of the free fraction of estrogen levels is associated with the initiation of thermoregulatory dysfunction in women [47]. It was reported that alleviation of hot flushes in women and in rats both require chronic treatment with estrogen (up to a month in women and 3–4 days in rats), suggesting that the action of estrogen is indirect and may involve a cascade in gene expression events including the expression of neurotransmitters and neuropeptides participating in thermoregulation such as serotonin [2]. After 3-day treatment, EEP phenolic compounds significantly decreased the number, the duration and the frequency of hot flushes probably by an ER-dependent mechanism as mentioned above.

In this study, EEP exhibited estrogen-like activity in vivo but seems to be antiestrogenic in vitro. Phytoestrogens is known to have mixed estrogen agonist/antagonist properties which are clinically useful [46]. The fact that EEP did not induced reporter gene activation in cell based assay, while it induced an estrogen uterotrophic response in vivo might either be due the high amount of active principles that induced “down regulation” of ERs in HEK293T cells or because active principles of EEP need some enzymatic transformation to be effective, given that in vivo assays take into consideration effects of metabolism, plasma-protein binding and pharmacokinetics [48]. Since a high increase in endometrium height could be a potential risk of endometrial cancer as seen in estrogen replacement therapy [49], the weak effects of EEP in uterine wall can be a safe alternative to HRT with minimal side effect. Vaginal epithelial proliferation and cornification observed with EEP treatment are desired estrogenic effects, because the lactobacillus use these superficial cells to produce lactic acid, which keeps the vaginal milieu acidic and thus prevent ascending infections [50]; Moreover, vaginal secretive cells will keep the vagina wet, thereby avoiding vaginal dryness. The Combination of therapy with bee venom (0.2 mg) and bee propolis (0.5 g) was shown to alleviate hot flushes in 85% of treated menopausal women [51]. These observations concord with part of results obtained with EEP (150 mg/kg) in hot flushes induced in rats.

## Conclusion

A universal standardization of propolis would be difficult because propolis biological actions should be linked to its chemical composition and plant sources. Ethanol extracted Cameroonian propolis has now been proven to possess estrogenic-like effects in rat. Knowing that estrogenic properties observed in estrogen-deficient animals following administration of plant preparations are associated to phytoestrogens actions, these results suggest that phenolic compounds detected in Cameroonian propolis may be a potential source of phytoestrogens. This might lead to its possible development as an improved traditional medicine for the alleviation of postmenopausal complaints following further investigation.

## Abbreviations

ACN: Acetonitrile
APPI: Atmospheric Pressure Photo Ionisation
BPI: Base peak intensity
CAPE: Caffeic acid phenethyl ester
DMEM: Dulbecco’s modified eagle’s medium
E2V: Estradiol valerate
EC: Elemental compositions
EEP: Ethanolic extract of Cameroonian propolis
ER: Estrogen receptor
ERE: Estrogen receptor element
ERα: Estrogen receptor alpha
ERβ: Estrogen receptor beta
ESI: Electrospray ionization
FA: Formic acid
FBS: Fetal bovine serum
FCS: Fetal calf serum
GC: Gas chromatography
HEK293T: Human Embryonic Kidney 233 T cells
HRMS: High-resolution Mass Spectrometry
HRT: Hormone Replacement Therapy
MS: Mass Spectroscopy
ONPG: O-nitrophenyl-β-galactopyranoside
OVX: Bilaterally ovariectomized rats
PRO: PROMAX-C
RDBeq: Ring Double Bond Equivalent
RPMI: Roswell Park Memorial Institute
Rt: Retention time
Sham: Sham operated animals
SEM: Standard Error of Mean
SRB: Sulforhodamine-B
UPLC: Ultra Performance Liquid Chromatography
